# Genome-wide identification and validation of tomato-encoded sRNA as the cross-species antifungal factors targeting the virulence genes of *Botrytis cinerea*

**DOI:** 10.3389/fpls.2023.1072181

**Published:** 2023-02-02

**Authors:** Fangli Wu, Yani Huang, Wenqin Jiang, Weibo Jin

**Affiliations:** Key Laboratory of Plant Secondary Metabolism and Regulation of Zhejiang Province, College of Life Sciences and Medicine, Zhejiang Sci-Tech University, Hangzhou, China

**Keywords:** tomato-derived sRNA, cross-species regulation, RNAi, virulence inhibition, B. cinerea

## Abstract

Recent evidence shows that small RNAs are transferred from a species to another through cross-species transmission and exhibit biological activities in the receptor. In this study, we focused on tomato-derived sRNAs play a role of defense against *Botrytis cinerea*. Bioinformatics method was firstly employed to identify tomato-encoded sRNAs as the cross-species antifungal factors targeting *B. cinerea* genes. Then the expression levels of some identifed sRNAs were checked in *B. cinerea*-infected plant using qRT-PCR method. Exogenic RNA-induced gene silences analysis were performed to investigate the antifungal roles of the sRNAs, and the target genes in *B. cinerea* of antifungal sRNAs would be confirmed by using co-expression analysis. Results showed that a total of 21 *B.cinerea*-induced sRNAs with high abundance were identified as the cross-kingdom regulator candidates. Among them, three sRNAs containing a miRNA (miR396a-5p) and two siRNA (siR3 and siR14) were selected for experimental validation and bioassay analysis. qRT-PCR confirmed that all of these 3 sRNAs were induced in tomato leaves by *B. cinerea* infection. Correspondingly, 4 virulence genes of B. cinerea respectively targeted by these 3 sRNAs were down-regulated. Bioassay revealed that all of these 3 cross-species sRNAs could inhibit the virulence and spore gemination of *B. cinerea*. Correspondingly, the coding genes of *B. cinerea* targeted by these sRNAs were also down-regulated. Moreover, the virulence inhibition by double strand sRNA was more effective than that by single strand sRNA. The inhibition efficiency of sRNA against *B. cinerea* increased with the increase of its concentration. Our findings provide new evidence into the coevolution of pathogens and host plants, as well as new directions for the use of plant-derived sRNAs to control pathogens.

## 1 Introduction

*Botrytis cinerea* represents one of the most predominant and common necrotrophic fungal pathogen promoting postharvest decay of fresh fruit and vegetables (Romanazzi and Feliziani, 2014). *B. cinerea* has a wide range of hosts and can infect over 200 plant species, causing grey mould disease (Schumacher, 2012; Kumar et al., 2020). Correspondingly, plants have also developed effective strategies against *B. cinerea* pathogen involving in two internal immune systems: PAMP triggered immunity (PTI) and effector triggered immunity (ETI). PTI and ETI mainly use proteins as action points, in which the microbial associated molecular pattern (MAMP) from pathogens or the damage associated molecular pattern (DMAP) from plants are used as triggers, and plant receptors are used as detectors (Duanis-Assaf et al., 2022). In addition, more and more evidence showed that ncRNA can be used as a mobile immune factor to antagonize the virulence of invasive filamentous pathogens (fungi and oomycetes) in a sequence dependent manner. In 2013, a groundbreaking work showed that the plant fungal pathogen *B. cinerea* could deliver sRNA to plant cells, and the mobile fungal sRNA could be loaded onto the plant silencing complex containing AGO1 to hijack plant immunity (Weiberg et al., 2013).

In contrary, expressing sRNAs targeting *B. cinerea* (Bc) *DCL* genes in plants results in successful silencing of the *BcDCL* genes, which in turn inhibits the generation of *B. cinerea* sRNAs that have been proven to be able to hijack plant immunity in a trans-kingdom manner (Wang et al., 2016). Cai et al. (2018) successfully transferred sRNA from *Arabidopsis thaliana* to *B. cinerea* through extracellular vesicles and then silenced fungus target genes *in vivo*. In addition, dsRNAs and small RNAs (sRNAs) targeting *DCL1* and *DCL2* of *B. cinerea* have been applied for disease control through RNA spraying (Wang et al., 2016; Wang et al., 2017; Meng et al., 2020; Qiao et al., 2021). Spraying a β2-tubulin dsRNA could conferred plant resistance to *B. cinerea* (Gu et al., 2019). Nerva et al. (2020) applied dsRNA targeting *BcCYP51*, *Bcchs1*, and *BcEF2* to suppress *B. cinerea* infection by high pressure spraying of grapevine leaves and postharvest spraying of grape bunches, and results indicate that RNA-based method to control *B.cinerea* is effective and environmentally friendly (Wang and Jin, 2017). Therefore, systematic identification of numerous RNAs, which could inhibit *B. cinerea* virulence infection, is important for understanding the resistance mechanism of plant against *B. cinerea* and applying RNA-based method to control *B. cinerea*. In this study, we report the discovery and validation of tomato-derived sRNAs in genome wide, which can inhibit the infection of *B. cinerea*. Our findings provide new evidence into the coevolution of pathogens and host plants, as well as new directions for the use of plant-derived sRNAs to control pathogens.

## 2 Materials and methods

### 2.1 Data sets

Eight sRNA-seq data sets produced from *B. cinerea*-infected tomato were downloaded from SRA database according to the accession numbers (GSM1101912, GSM1101913, GSM1101914, GSM1101915, GSM1101916, GSM1101917, SRR1482408 and SRR1463412). Among them, GSM1101912, GSM1101914 and GSM1101915 were respectively produced from the *B.cinerea*-inoculated leaves at 0h, 24 h and 72 h by Weiberg et al. (2013), whereas SRR1482408 and SRR1463412 were produced from the mock- and *B.cinerea*-inoculated tomato at 7 days (Jin and Wu, 2015). Tomato genome sequences were downloaded from ftp://ftp.solgenomics.net/(version:build_2.40). The datasets of virulence factors in fungal pathogens were downloaded from http://sysbio.unl.edu/DFVF/index.php (Lu et al., 2012).

### 2.2 Systemic identification of tomato-derived sRNAs targeting *B. cinerea*

To identify *B. cinerea*-inducted sRNA from tomato, two sRNA-seq datasets which were sequenced from *B. cinerea*-inoculated (TD7d) and mock-inoculated (TC7d) tomato leaves at 7 days post-inoculation (dpi) were downloaded from NCBI SRA database with the accession number SRP043615 (Jin and Wu, 2015). The unique sequences in each library were extracted and combined into 1 sRNA library for sRNA identification; all reads in both libraries that were exactly mapped to tomato genomic sequences but unmapped to tRNA, rRNA and *B.cinerea* genome sequence were extracted as tomato sRNA. The raw read count of these unique sRNAs were retrieved from each sRNA libraries and normalized to reads per millions (RPM). The sRNA sequences with a minimum RPM of 10 in each library were extracted for different expression analysis. The upregulated sRNAs with the Log2(TD7d/TC7d) of >1 and and p-value <0.001 in *B. cinerea*-infected tomato were considered as the candidates of *B. cinerea*-induced sRNAs in tomato. Then these candidates were confirmed by sRNA-seq datasets produced from *B.cinerea*-inoculated tomato at 24 hpi and 72 hpi comparing with mock-inoculated tomato by Weiberg et al. (2013). These *B.cinerea*-induced sRNAs were considered as miRNAs through sequences alignment with known miRNAs deposited in miRBase database (Kozomara and Griffiths-Jones, 2011). The remaining sRNAs were considered as unknown siRNA. The target genes of the identified sRNA including miRNAs and siRNAs were predicted by using psRNATarget server against cDNA sequences of *B. cinerea* (Dai and Zhao, 2011). The virulence factors were analyzed for the putative targets by using blastp with the parameters of E-value <1e-5, identity >40% and coverage >70%.

### 2.3 Preparation of plant materials and inoculation of *B. cinere*

The seeds of tomatoes cv. *Micro Tom* and *Nicotiana benthamiana* were seeded directly into the soil with a 12h:12h photoperiod at ~22°C in a greenhouse. This work used 6-week-old plants. Potato dextrose agar was used for the cultivation of *B. cinerea*. Conidiospore has collected from 2-week-old infected tomato washed with distilled water two times and adjusted the concentration of 5 × 10^6^ conidiospores/mL for bioassay. Based on the sequence shown in **Table S2**, both single strand (ss) sRNAs and double strand (ds) sRNAs were synthesized by GenePharma (Shanghai, China). Each sRNA was added to the conidiospore suspension at a final concentration of 10 µM and immediately drop-inoculated onto five tobacco leaves or tomato leaves for several days. The leaves in control were inoculated with conidiospores in the same manner but with water or negative control RNA (NC RNA, 5′-UUCUCCGAACGUGUCACGUTT-3′. has no target gene in *B. cinerea*). The experiment was repeated three times. We analyzed the intensity of infection of *B. cinerea* control and treatment with sRNA by imaging and Trypan blue staining (Woods-Tör et al., 2018).

### 2.4 Conidiospore germination assays

Conidiospore germination was detected using the cellophane strip method described by Bilir et al. (2019). Briefly, cellophane strips were cut into 1.5 cm2, sterilized by high pressure, and then placed onto MS media in Petri dishes and were added with 10 µL of *B. cinerea* conidiospore with sRNA or NC-RNA treatment. After incubating for 12 h:12 h photoperiod at 22°C, conidiospores were examined under a light microscope.

### 2.5 Total RNA extraction and quantitative RT-PCR (qRT-PCR)

Total RNAs were extracted using TRNzol-A+ reagent (TIANGEN, Beijing, China), and a Nano Drop 2000 spectrophotometer was used for RNA quantification. The steps of reverse transcription for the mRNA and sRNA are different. For mRNAs, reverse transcription was performed using the PrimeScript RT Reagent Kit with gDNA Eraser (Takara, Dalian, China) according to the manufacturer’s recommendations. A similar reaction without reverse transcriptase was performed as a control to confirm the absence of genomic DNA in subsequent steps. For miRNAs, we added poly(A) using *E. coli* poly(A) polymerase (NEB, Beijing, China). 3’ RT-Primer (Invitrogen) was used as the reverse transcription primer for the following reverse transcription according to the manufacturer’s protocol.

SYBR Green PCR was performed in accordance with the manufacturer’s instructions (NEB, Beijing, China). In brief, 1 μL of cDNA template was added to 10 μL of 2X Master Mix (NEB, Beijing, China), and 10 μM of specific primers and ddH_2_O were added to a final volume of 20 μL. The reaction was pre-denatured for 3 min at 94 °C, followed by 50 cycles of 94°C for 30 s and 58°C for 30 s. All reactions were performed in triplicate, and controls (no template) were included for each gene. Threshold cycle (Ct) values were automatically determined using the ABI 7500 Real-Time PCR System (USA). The confirmation of amplicon specificity was based on the melt curve at the end of each run. Fold changes were calculated using the 2^-ΔΔCt^ method, where 2^-ΔΔCt^ = (Ct, target − Ct, inner) Treatment − (Ct, target – Ct, inner) Control (Livak and Schmittgen, 2001). All oligos used in this study are listed in **Supplementary Table S5**.

### 2.6 Vector construction and co-expression of the target with miR396a

Pre-miR396a was synthesized and then cloned into the pBIN438 vector with BamH I and Sal I. The cDNA sequences of *B. cinerea* genes targeted by miR396a were obtained using the RT-PCR method and then inserted into the pEarleyGate 100 expression vector down-stream of the CaMV 35S promoter region. The target sites of miR396a-5p in the target genes were deleted using overlap PCR. Subsequently, the mutated sequences were also inserted into the pEarleyGate 100 vector with expression driven by CaMV 35S promoter.

Transient expression experiments were performed in accordance with the method described by Meng et al. (2020). The *Agrobacterium tumefaciens* strain GV3101 was transformed with the constructs p35S::MIR396a, p35S::target (normal target site), and p35S::target^mu^ (target site deletion). Transformed *A. tumefaciens* samples were harvested and resuspended in infiltration medium [10 mM MgCl2, 10 mM MES (pH 5.6), 200 μM acetosyringone] with the OD600 adjusted to approximately 0.2 for 3-4 h. The *A. tumefaciens* was infiltrated into 2-week-old *N. benthamiana* leaves, which were harvested 2 days later.

### 2.7 Statistical analysis

The statistical analysis was performed with SPSS statistical software 22.0 (United States). All the results were expressed as the Means with SDs from three independent experiments. The *t*-test was selected and the *P*-values < 0.05 were considered statistically significant.

## 3 Results

### 3.1 Genome-wide identification of tomato-derived sRNAs targeting *B. cinerea*

Two sRNA-seq datasets were sequenced from *B. cinerea*-inoculated (TD7d), and mock-inoculated (TC7d) tomato leaves at 7 days post-inoculation (dpi) were downloaded from the NCBI SRA database with the accession number of SRP043615 to identify tomato-derived sRNAs targeting *B. cinerea* (Jin and Wu, 2015). By using 10 normalized RPM sRNA reads as a cutoff, a total of 1373 sRNAs, ranging from 20 nt to 24 nt, were identified in both libraries (**Table S1**). Among them, 53 sRNAs were upregulated in *B. cinerea*-infected leaves compared with mock-infected leaves and then considered as *B. cinerea*-induced sRNA candidates (**Table S2**). Moreover, 21 out of the 53 sRNAs were upregulated in *B. cinerea*-inoculated tomato libraries reported by Weiberg et al. (2013) (**Table S2**, **Table 1**), but the remaining 32 sRNAs could not be confirmed (**Table S2**). Of these 21 expression-confirmed sRNAs, six had been reported as miRNAs in miRBase, and the remaining 15 were unknown, which were labeled as siR1–siR15 (**Table 1**). In addition, six *B. cinerea*-induced sRNAs including 2 miRNAs (miR156d-5p and miR396a-5p) and four siRNAs (siR3, siR10, siR13, and siR14) were confirmed by two or more *B. cinerea-*treated datasets reported by Weiberg et al. (2013) (**Table 1**).

**Table 1:** Confirmation of *B.cinerea*-induced sRNA in tomato by sRNA-seq datasets produced from *B.cinerea*-inoculated tomato at 24 hpi and 72 hpi comparing with mock-inoculated tomato by Weiberg et al. (2013).

sRNA_id*	annotation	sRNA_Name	Confirmation in different dataset			
			24hpi_leaf *vs*. Mock	72hpi_leaf *vs*. Mock	24hpi_fruit *vs*. Mock	72hpi_fruit *vs*. Mock
s100001	ath-miR157a-5p	miR157a_5p			Up	
s100006	sly-miR156d-5p	miR156d_5p			Up	Up
s100033	sly-miR5300	miR5300			Up	
s100063	Unknown siRNA	siR1		Up		
s100120	Unknown siRNA	siR2				Up
s100182	Unknown siRNA	siR3	Up	Up		
s100241	sly-miR396a-5p	miR396a_5p	Up		Up	
s100282	Unknown siRNA	siR4		Up		
s100407	Unknown siRNA	siR5			Up	
s100439	sly-miR396a-3p	miR396a_3p			Up	
s100544	Unknown siRNA	siR6	Up			
s100841	sly-miR482b	miR482b		Up		
s100916	Unknown siRNA	siR7	Up			
s101071	Unknown siRNA	siR8				Up
s101117	Unknown siRNA	siR9			Up	
s101225	Unknown siRNA	siR10	Up		Up	
s101249	Unknown siRNA	siR11			Up	
s101279	Unknown siRNA	siR12				Up
s101316	Unknown siRNA	siR13	Up		Up	Up
s101327	Unknown siRNA	siR14		Up		Up
s101332	Unknown siRNA	siR15				Up

*the sRNA sequence can be retrieved from **Table S1** or **Table S2** according to **sRNA_id**.

We identified potential target genes of these 21 *B. cinerea*-induced sRNAs in silico and focused on those associated with fungi virulence factors to understand the roles of these sRNAs in cross-kingdom. The results showed that a total of 149 transcripts of *B. cinerea* were identified as putative targets by psRNAtarget with default parameters (http://plantgrn.noble.org/psRNATarget/) (**Table S3**). By searching the database of virulence factors in fungal pathogens, we revealed that 82 transcripts targeted by the 21 sRNAs shared high sequence similarity with 63 virulence factors, indicating their potential roles in the pathogenicity of *B. cinerea* (**Table S4**). Moreover, 13 transcripts were targeted by nine sRNAs, namely, miR396a-5p, miR482b, siR2, siR3, siR5, siR8, siR9, siR14, and siR15, which were involved in “Grey mould” (**Table 2**). The transcripts were targeted by the remaining 12 sRNAs, namely, miR157a-5p, miR156d-5p, miR5300, miR396a_3p, siR1, siR4, siR6, siR7, siR10, siR11, siR12, and siR13, which were reported as virulence factors in other fungal pathogens.

**Table 2:** The target genes of sRNAs were related with Grey mould.

sRNA_name	Taget proteins	Annotation	UniProtID	Gene Symbol
miR396a_5p	Bcin15p02590.2	adenylate cyclase	Q9P880_BOTFU	BAC
miR396a_5p	Bcin04p04920.1	thiamine biosynthesis protein	O94101_BOTFU	BCNMT1
miR482b	Bcin09p01310.1	neutral trehalase protein	Q156F4_BOTFU	NULL
miR482b	Bcin09p02390.1	cmgc mapk protein kinase protein	Q000T6_BOTFU	BMP3
miR482b	Bcin11p04600.1	mfs transporter protein	Q9P8L8_BOTFU	BCMFS1
siR2	Bcin05p02840.1	phosphoinositide-specific phospholipase C (BCPLC1)	P78585_BOTFU	BCPLC1
siR3	Bcin04p03120.1	putative chitin synthase g protein	Q8TG14_BOTFU	BCCHSIII
siR5	Bcin08p02970.1	pectin methylesterase protein	Q9C2Y1_BOTFU	BCPME1
siR8	Bcin13p01840.1	Colletotrichum karsti peptidyl-prolyl cis-trans isomerase 10 (CkaCkLH20_04771), partial mRNA/unknown	Q6WP53_BOTFU	BCP1
siR9	Bcin15p02590.2	putative adenylate cyclase	Q9P880_BOTFU	BAC
siR12	Bcin01p06260.6	two-component osmosensing histidine kinase BOS1	Q8X1E7_BOTFU	BOS1
siR14	Bcin01p06260.1	two-component osmosensing histidine kinase BOS1	Q8X1E7_BOTFU	BOS1
siR15	Bcin03p06840.1	nadph oxidase regulator protein	B0BER8_BOTFU	NOXR
siR15	Bcin05p00350.1	nadph oxidase protein	B0BES1_BOTFU	NOXA
siR15	Bcin09p06130.1	tetraspanin protein	Q8J0E3_BOTFU	PLS1

### 3.2 Expression patterns of *B. cinerea*-induced sRNAs and their target genes

**Figure 1:**
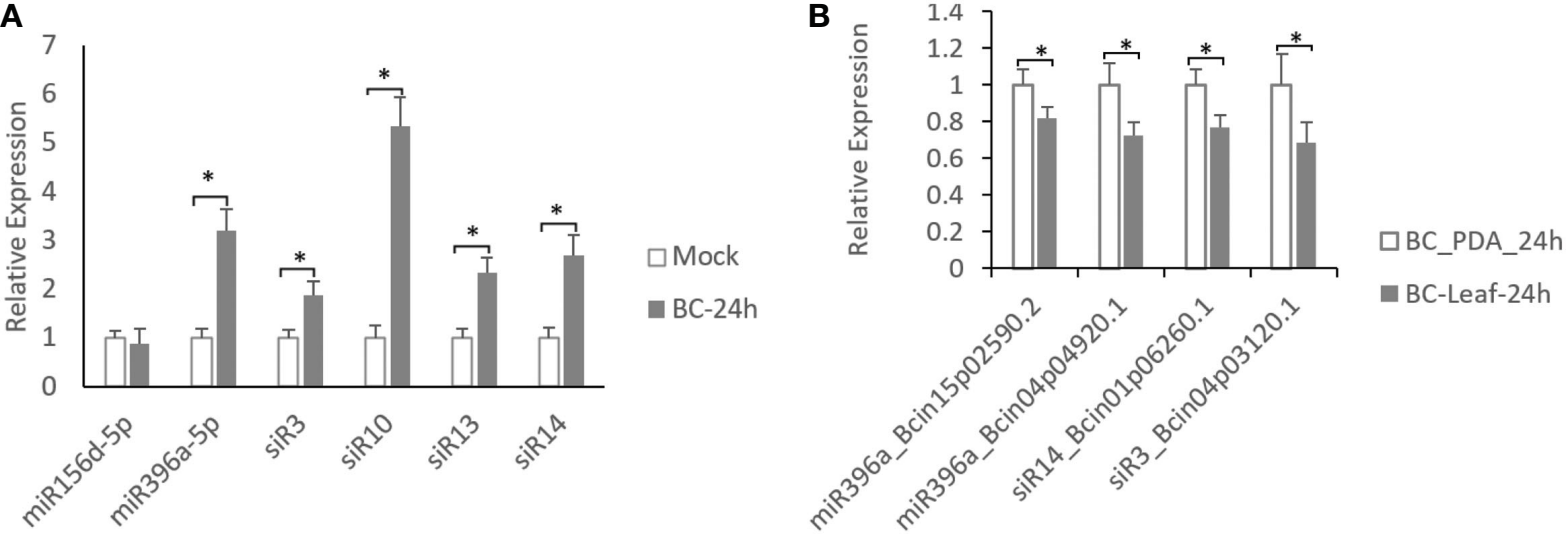
Expression analysis of *B.cinerea*-induces sRNAs and their targets. **(A)** Expression levels of sRNAs in *B cinerea*-inoculated leaves compared with mock-inoculated leaves in tomato. **(B)** Expression levels of 4 coding genes targeted by 3 sRNAs in *B cinerea* grew on tomato leaves comparted with that grew on PDA plates. Asterisks indicate a significant difference (^∗^P < 0.05).

To confirm the expression of these identified sRNAs induced by *B. cinerea*, 6 sRNAs (miR156d-5p, miR396a-5p, siR3, siR10, siR13 and siR14) supported by two or more other *B. cinerea*-treated datasets were selected to measure their expression patterns in *B. cinerea*-inoculated tomato leaves at 24 hours post-inoculation (hpi) using quantitative reverse transcription PCR (qRT-PCR). Results showed that all of these sRNAs were upregulated in *B. cinerea*-infected tomato leaves compare with mock-treated leaves except for *miR156d-5p* (**Figure 1A**), showing a consistent expression pattern with sRNA-seq datasets (Weiberg et al., 2013; Jia et al., 2015). Of these 5 *B. cinerea*-induced sRNAs, only 3 sRNAs might target four virulence factor coding genes of grey mould disease, including *Bcin15p02590.2* and *Bcin04p04920.1* targeted by *miR396a-5p*, *Bcin04p03120.1* targeted by *siR3* and *Bcin01p06260.1* targeted by *miR14* (**Table 2**). Therefore, expression patterns of these 4 coding genes were also investigated in *B. cinerea* inoculated on the potato leaves comparted with that on the the PDA plates the by using qRT-PCR. Results showed that all of these four targets were down-regulated (**Figure 1B**), showing a negative regulation with their sRNAs.

### 3.3 Antifungal activities of *B. cinerea*-inducted sRNAs

**Figure 2:**
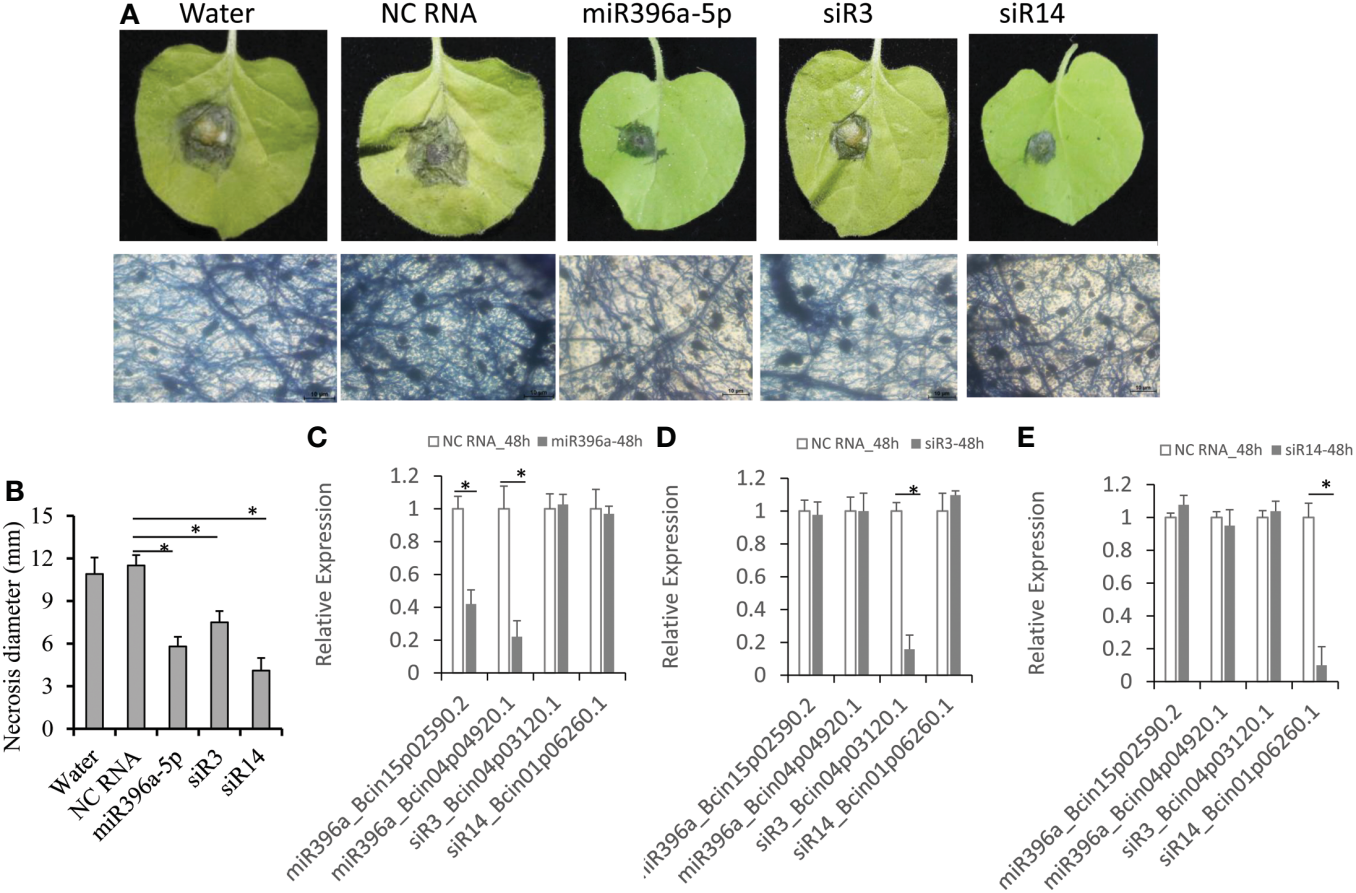
Antifungal activities of tomato-derived sRNAs. **(A)** Tobacco leaves were inoculated with *B cinerea* conidiospores containing tomato-derived sRNAs. **(B)** Diameters of the necrosis on tobacco leaves. **(C–E)** Expression analysis of 4 coding genes targeted by the 3 sRNAs in *B cinerea* treated with miR396a-5p, siR3 and siR14, respectively. Results are expressed as means ± SD of 3 biological replicates. Asterisks indicate a significant difference (*P<0.05) compared to the corresponding NC-RNA treatment.

Evidences showed that exogenous RNA-induced gene silence (ERIGS) is an efficient method to control grey mould disease by adding *B. cinerea* gene-targeted sRNAs (Wang et al., 2016; Wang et al., 2017; Meng et al., 2020; Qiao et al., 2021). Therefore, we used ERIGS to test the antifungal activities of these 3 cross-species sRNAs against *B. cinerea*. These three cross-species sRNAs (miR396a-5p, siR3 and siR14) were added separately into the *B. cinerea* conidiospore solution to a final concentration of 10 μM and then drop-inoculated to at least six tobacco leaves for 3 days. Result showed that the average diameter of necrosis reached ~11 mm on *B. cinerea*-inoculated leaves with water or NC RNA treatments (**Figures 2A, B**). On the leaves of sRNA-treated *B. cinerea*, three sRNAs had smaller necrosis than those mock treated (**Figure 2A**). Among them, siR14 hardly had a necrotic diameter (4.1 mm), followed by miR396a-5p (5.8 mm), and the remaining siR3 had a similar necrotic diameter (~7.5 mm, **Figure 2B**). Correspondingly, the virulence factors coding genes (*Bcin15p02590.2* and *Bcin04p04920.1*) targeted by miR396a-5p were significantly down-regulated in *B. cinerea* treated by miR396a-5p, but no expression changes in *B. cinerea* treated by the other two sRNAs (**Figures 2C–E**). Similarity, the expression of *Bcin04p03120.1* targeted by siR3 and *Bcin01p06260.1* targeted by siR14 were only down-regulated in *B. cinerea* treated by siR3 and siR14, respectively (**Figures 2C–E**). These results suggesting that these 3 sRNAs control grey mould disease by targeting the virulence genes of *B. cinerea*.

### 3.4 sRNAs inhibits spore germination

**Figure 3:**
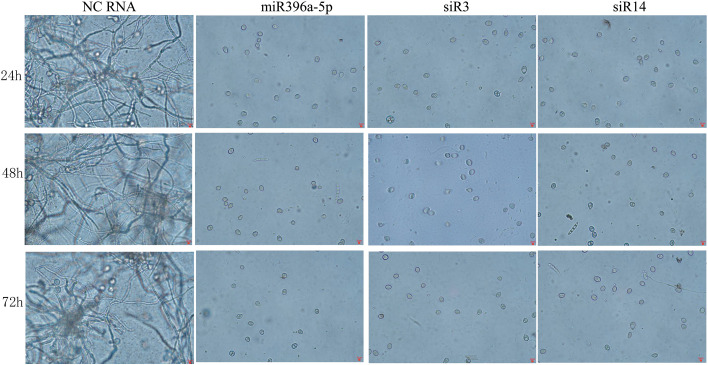
Germination assays of *B cinerea* conidiospores treated by tomato-derived sRNAs.

Evidences showed that adding exogenous sRNAs can inhibit the spore germination and hence infection of pathogenic fungi by inducing RNAi pathway (Bilir et al., 2019; Meng et al., 2020). Therefore, Germination assays were performed at 24 h, 48 h and 72 h to understand the effects of the 3 sRNAs on the conidiospore germination of *B. cinerea*. Results showed that most of the spores incubated with NC-RNA germinated successfully and the mycelia grew well within 24 hours, but the spores treated with each of these 3 sRNAs (miR396a-5p, siR3 and siR14) failed to germinate (**Figure 3**). These results suggest that RNA has a significant inhibitory effect on spore germination of *B. Cinerea.*

### 3.5 Effects of sRNA concentration on antifungal activities

**Figure 4:**
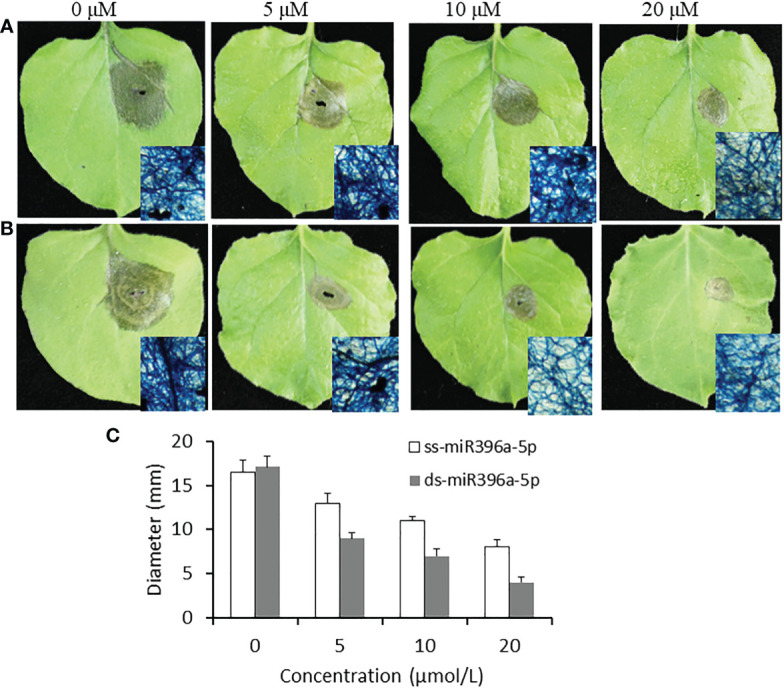
Effect of miR396a-5p concentration on the virulence inhibition. A) Tobacco leaves were inoculated with *B cinerea* conidiospores containing 5 μM, 10 μM and 20 μM ss-miR396a-5p. Partial necrosis were stained by trypan blue method. B) Tobacco leaves were inoculated with *B cinerea* conidiospores containing 5 μM, 10 μM and 20 μM ds-miR396a-5p. Partial necrosis were stained by trypan blue method. C) Diameters of the necrosis on tobacco leaves. Results are expressed as means ± SD of 3 biological replicates.

**Figure 5:**
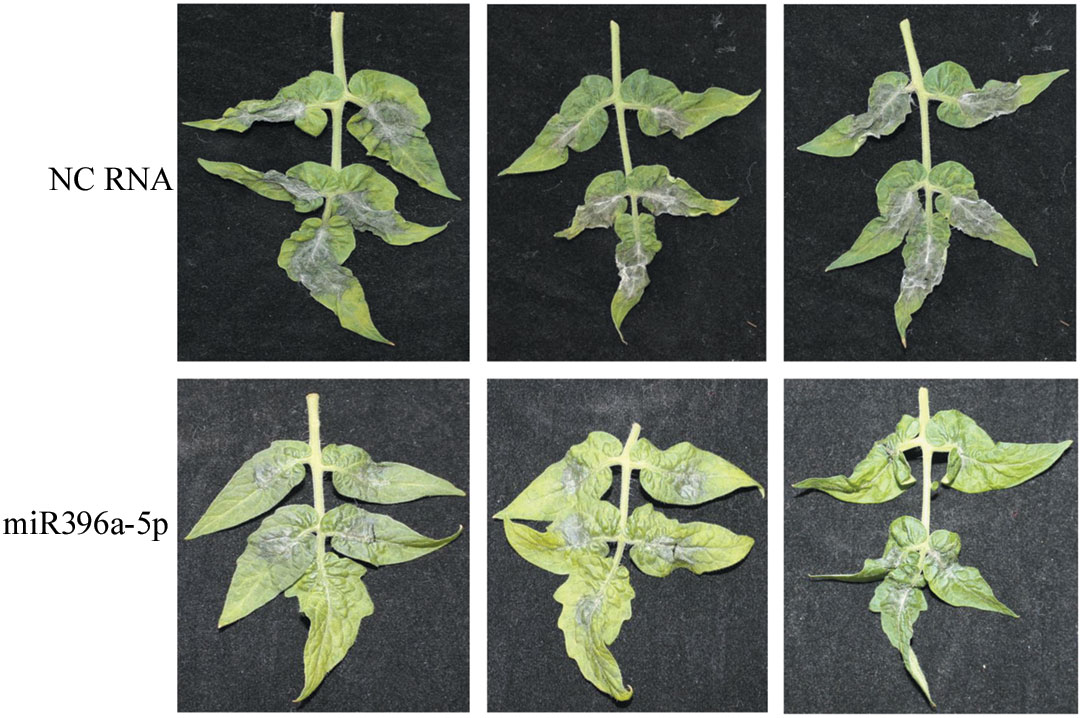
Antifungal activities of miR396a-5p in tomato leaves with three branches. Tomato leaves were inoculated with *B cinerea* conidiospores containing 20 μM ds-miR396a-5p and NC RNA.

miR396a-5p which has a medium antifungal activity in all of three sRNAs was selected in the subsequent assay for the effects of sRNA concentration. miR396a-5p was added to the conidiospore solution of *B. cinerea* with a final concentration of 5, 10 and 20 μM, and then inoculated to tobacco leaves for 4 days. For the single stand sRNA, miR396a-5p with 5μM has a largest necrosis diameter (13 mm); followed by 10μM; miR396a-5p treated with 20 μM has a minimal necrosis (8 mm) (**Figures 4A, C**). These results indicated that high concentration of sRNA has better anti-*B.cinerea* activity. A similar result was observed for the double strand miR396a-5p (**Figure 4B, C**). These results showed that the suppression by double strand miR396a-5p (ds-miR396a-5p) was more efficient than that by single strand miR396a-5p (ss-miR396a-5p) (**Figure 4C**). In addition, the antifungal activity of ds-miR396a-5p were also investigated on tomato leaves through inoculating conidiospore solution of *B. cinerea* with 10 uM ds-miR396a-5p. Results showed that the necrosis area of *B. cinerea* were significantly reduced in ds-miR396a-5p-treated leaves compared with that in NC RNA-treated leaves (**Figure 5**), exhibiting strong antifungal activity.

### 3.6 Validation of *B. cinerea* genes targeted by miR396a-5p

**Figure 6:**
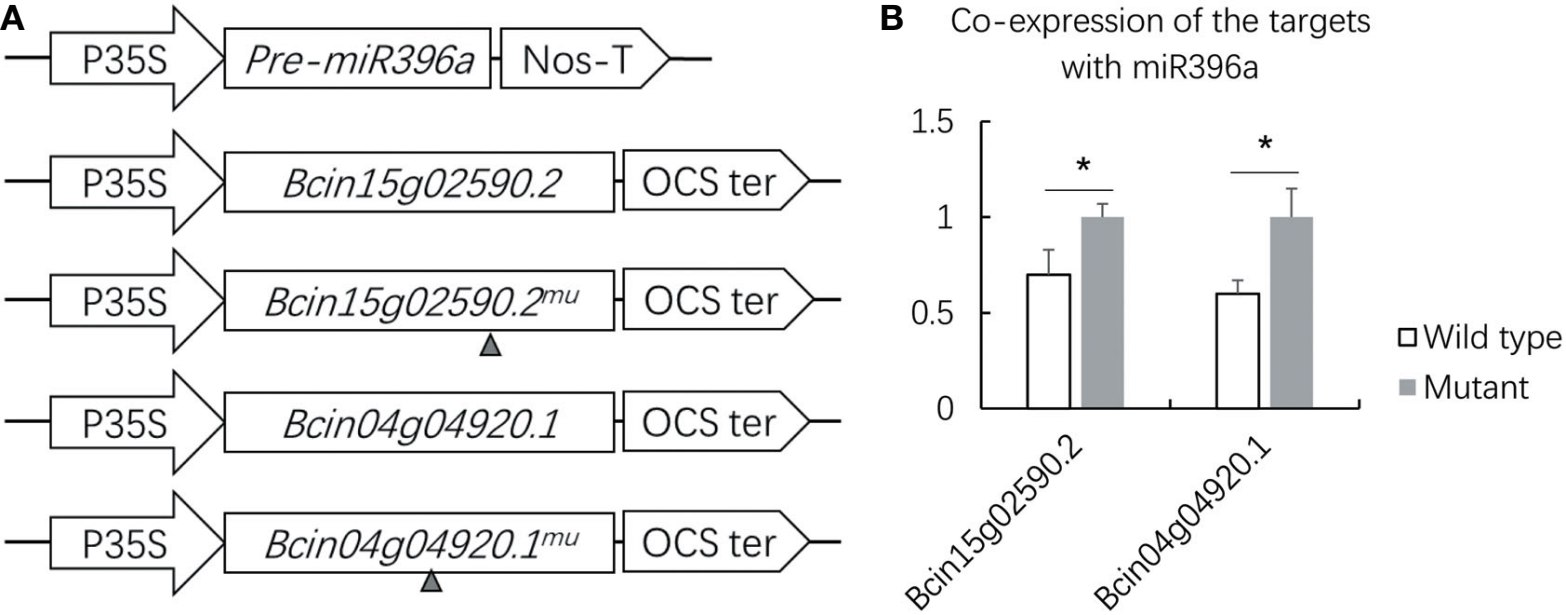
Validation of target gene of miR396a by transient co expression. **(A)** Overexpression vector constructs of miR396a-5p and its 2 target genes in *B cinerea* (*Bcin15g02590.2* and *Bcin04g04920.1*). Gray small triangle: refers to the deletion of the target site. **(B)** Relative transcription levels of the 2 target genes co-expressed with miR396a-5p in *N. benthamiana*. Results are expressed as means ± SD of 3 biological replicates. Asterisks indicate a significant difference (*P < 0.05) compared to the corresponding the transient expression of the wild type target gene.

To understand the anti-*B. cinerea* mechanism, transient co-expression was performed to validate two *B. cinerea* coding genes targeted by miR396a-5p which was predicted by psRNAtarget (**Table 2**, **Table S3**). We firstly constructed two types of the target gene vectors, which contained either a target site or lacked a target site, to further validate the identified targets of miR396a-5p (**Figure 6A**). target constructs together with the pre-miR396a were transiently co-expressed in *N. benthamiana*. As the control, each of two wild type target constructs was also transiently expressed in *N. benthamiana*. Compared with transient expression of the control, the relative expression levels of *Bcin15g02590.2* and *Bcin04g04920.1* without miR396a-5p target sites were not repressed by co-expression with pre-miR396a (**Figure 6B**). However, the transcript levels of *Bcin15g02590.2* and *Bcin04g04920.1* containing a target site were significantly decreased when co-expressed with pre-miR396a (**Figure 6B**). The results suggested that both virulence genes of *B. cinerea* could be negatively regulated by miR396a.

## 4 Discussion

Emerging evidence supports that RNAs can be transmitted as cross-species effectors from a low trophic level of the food chain and exhibit biological activities at a high trophic level (Jiang et al., 2012; Han and Luan, 2015; Zhang et al., 2012; Zhang H et al., 2016; Zeng et al., 2019). In addition to the study on the sRNAs transferred from a high trophic level to a low trophic level of the food chain and exhibit biological activities, Zhang T et al. (2016) found that cotton-derived miRNAs target *V. dahliae* virulence genes. The same phenomenon was found in our previous research that tomato-derived miR1001 can cross-species inhibit the growth and virulence of *B. cinerea* (Meng et al., 2020). In this study, we performed a genome-wide identification to screen the tomato-derived sRNAs acting as the cross-species inhibitors in the plant against *B. cinerea*. A total of 21 sRNAs were identified as the candidates of cross-species inhibitors. Of them, 3 tomato-derived sRNAs, namely miR396a-5p, siR3 and siR14, showed a role of the cross-species regulation of *B. cinerea* virulence. For these 3 sRNAs, a total of four virulence genes were predicted as the targets including adenylate cyclase, thiamine biosynthesis protein, chitin synthase and two-component histidine kinase (**Table 2**). Evidence that the mutants of adenylate cyclase (Liu et al., 2012), thiamine biosynthesis protein (Liu et al., 2022), chitin synthase (Zhang et al., 2016) and two-component histidine kinase (Zhang et al., 2010) would significantly reduce the pathogenicity of the pathogens. Therefore, we proposed that these 3 sRNAs inhibited the pathogenicity by targeting the virulence genes of *B. cinerea*. In addition, we also investigate the roles of endogenous genes in plant targeted by these 3 antifungal sRNAs (**Table S6**). Results showed that a large number of the coding genes can be targeted by these 3 sRNA in tomato, and most of these endogenous targets are related to plant growth and development and stress tolerances. For example, a total of 178 coding genes were predicted as the targets of miR396a-5p. previous study showed that miR396a is a plant conserved mRNA, which mainly regulates the growth and development of plant leaves by targeting GRF family members. Moreover, miR396a could also regulate the expression of *bHLH74*, participating to regulate the development of leaf margin shape and roots (Chen et al., 2015; Chen et al., 2017; Chandran et al., 2019). Interestingly, studies have found that overexpression of miR396a reduced the resistance of plant against to pathogens (Chen et al., 2015). In this study, we found that the exogenous application of miR396a can suppress the virulence of *B. cinerea*. Therefore, we speculate that the expression of pathogenic sRNAs in plants induced by *B. cinerea* is to help them successfully infect the host plants. On the contrary, in order to counter this invasion mode of pathogens, host might transport these induced pathogenic sRNAs into pathogens to weaken the role of pathogenic sRNAs in plant and inhibit the virulence of pathogens.

We consider that low-abundance sRNAs hardly play their role in cross-species gene regulation because they are difficult to transport to receptor species or the abundance in the receptor species is excessively low to play a corresponding role. Jia et al. (2015) found that the mulberry miR166b in silkworm is nonfunctional in cross-kingdom gene regulation. Lin et al. (2019) also mentioned that the abundance of cross-kingdom miRNAs detected in receptor species should correlate with the miRNA abundance in the original species unless a particular miRNA is highly stable or preferentially absorbed in animals (Zhang et al., 2012). However, the read count of miR162a is relatively high in *H. larvae* and shows a significant effect on cross-kingdom gene regulation (Zhu et al., 2017). Basing on this notion and sRNA-seq data, we selected the tomato-derived sRNA candidates with a minimum RPM of 10 in tomato, corresponding to the raw reads of >140.

Bilir et al. (2019) showed that exogenous single-strands sRNAs (ss-sRNAs) and double-strand sRNAs (ds-sRNAs) can be absorbed by spores and combined with target genes to form RNA complexes and inhibit the expression of the target genes. Ds-sRNA is more effective than ss-sRNA. A similar phenomenon was found in this study. When ss-miR396a-5p or ds-miR396a-5p was combined with the spores of *B. cinerea*, both sRNAs can inhibit the infection of *B. cinerea*. Moreover, ds-sRNA was more effective than ss-sRNA. The reason may be that ds-sRNA has higher binding efficiency with RISC than ss-sRNA. Martinez et al. (2002) revealed that RISC formation is undetectable at lower concentrations of ss-sRNA but can be detected even at 1 nmol/L concentration of ds-siRNA. Holen et al. (2003) showed that an excess of ds-siRNA can competitively block ss-sRNA. Lima et al. (2009) found that siRNA was more efficient than ssRNA in binding Dicer.

## Data availability statement

The original contributions presented in the study are included in the article/**Supplementary Material**. Further inquiries can be directed to the corresponding author.

## Author contributions

FW and WBJ conceived and designed the research. FW, YH and WQJ organized and performed the experiments. FW and WBJ analyzed the data and wrote the manuscript. All authors contributed to the article and approved the submitted version.
